# Innovative approaches to therapeutic target discovery amid the global challenge of antimicrobial resistance

**DOI:** 10.1007/s10822-026-00905-3

**Published:** 2026-07-30

**Authors:** Thayssa de Oliveira Teixeira, Ruana Carolina Cabral da Silva, Maria Cidinaria Silva Alves, Rousilândia de Araujo Silva, José Eduardo Souza Echeverria, Simone Simionatto

**Affiliations:** 1 Health Sciences Research Laboratory, Federal University of Grande Dourados (UFGD), Rodovia Dourados – Itahum, km 12, Cidade Universitária, Dourados, Mato Grosso do Sul 79804970 Brazil; 2 Barretos Cancer Hospital, São Paulo, Brazil; 3 Department of Pharmaceutical Sciences, Federal University of Pernambuco (UFPE), Recife, PE Brazil

**Keywords:** Structural modeling, Protein function prediction, Bioinformatics, Multidrug-resistant bacteria, Artificial intelligence

## Abstract

Antimicrobial resistance is a constant threat to global public health, requiring innovative strategies for therapeutic target identification. Hence, this narrative review discusses the application of structural modeling and artificial intelligence in the functional prediction of proteins encoded by multidrug-resistant bacterial genomes. Tools such as AlphaFold and RoseTTAFold have enabled high-accuracy three-dimensional structure prediction, facilitating the annotation of hypothetical proteins and the identification of conserved domains and catalytic sites. These computational approaches bridge the gap between genomic data and biological function, accelerating drug discovery and guiding design of new antimicrobial bioactive compounds. Despite notable advances, several challenges have persisted regarding experimental validation and genomic variability, revealing an opportunity to integrate artificial intelligence-driven modeling with bioinformatics as a transformative method for better understanding resistance mechanisms and prioritizing novel therapeutic targets.

**Graphical abstract:**

**Figure:**
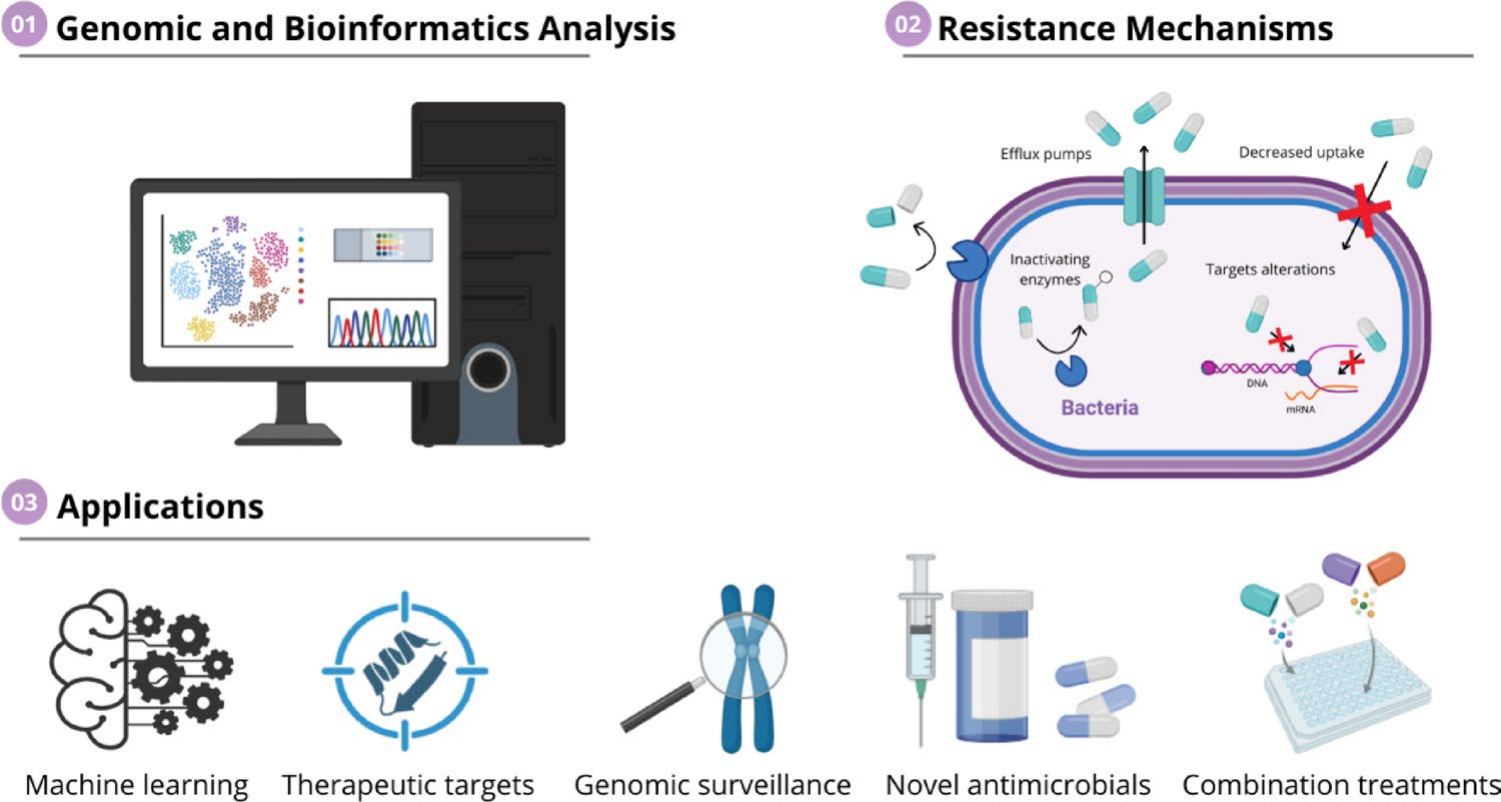
This review highlights how artificial intelligence bridges bacterial genomics and antimicrobial drug discovery. Its relevance stems from the validation of computational approaches that transcend the constraints of conventional lab-based biology, allowing for the pinpoint identification of catalytic sites in resistant strains. By outlining the current landscape and validation hurdles, this study offers a strategic framework for the fast-tracked, cost-effective prioritization of therapeutic targets, making it a vital resource for tackling emerging pathogens.

## Introduction

Antimicrobial resistance (AMR) has progressively intensified, constituting a significant threat to global public health. Recognized by the World Health Organization as one of the greatest health challenges of our time, AMR was associated with approximately 4.95 million deaths in 2019, of which 1.27 million were directly attributable to resistant bacterial infections. One of the main factors contributing to this scenario is the inappropriate use of antibiotics, which promotes natural selection and persistence of resistant microorganisms, thereby compromising the efficacy of available treatments [1].

The selective pressure exerted by a single antimicrobial agent can foster the emergence of bacterial variants that not only resist that agent but may also acquire resistance to multiple drugs. The emergence of such multidrug-resistant pathogens significantly reduces the effectiveness of currently available therapies for infection treatment [2]. Despite the approval of approximately 40 new antibiotics by the Food and Drug Administration since 2000, most utilize previously established mechanisms of action. Coupled with high costs and lengthy development time, this constraint hampers the introduction of truly innovative therapies, even when urgent medical needs exist [3].

In this context, computational strategies have gained prominence due to their potential to accelerate the identification and functional characterization of proteins, a crucial step toward expanding the therapeutic target repertoire and enabling innovative strategies against resistant microorganisms. Nevertheless, a substantial gap persists between the rapidly growing number of proteins identified via genomic sequencing and our effective understanding of their biological functions. Accordingly, inferring protein function remains one of the major challenges in modern computational biology [4].

Overcoming this challenge has driven the development of more design of new bioactive compounds approaches, grounded in an increasingly detailed understanding of the molecular mechanisms underlying infectious diseases. A critical step in this process is identifying specific residues responsible for protein function, as such information enables precise structure–activity relationship analyses. These insights allow researchers to modify or optimize bioactive molecules (e.g., inhibitors or activators) to enhance their interaction with specific therapeutic targets, thus enhancing the selection of promising compounds and contributing to the development of more effective antimicrobial agents [5–7]. Rapid advances in computational technologies now enable simulation of complex biological and chemical systems at unprecedented levels of detail. Modern graphics processing units can significantly enhance computational chemistry applications, including molecular dynamics simulations of biomolecules, providing valuable insights into conformational changes and protein–ligand or protein–protein interactions that underpin biological function [5].

Furthermore, artificial intelligence (AI)-driven structure prediction has emerged as a potential ally in protein functional inference. Several methods have been shown to leverage structural information to identify functional regions, and these approaches have recently been substantially improved through advances in deep learning [7, 8]. Computational tools have demonstrated highly accurate prediction of three-dimensional structures, achieving quality comparable to experimentally determined models [9]. Consequently, it is now possible to generate detailed structural models for virtually all sequenced proteins [7]. Such knowledge is essential for developing and optimizing bioactive compounds with desirable properties [5].

Given this scenario, this review aimed to compile and discuss some of the main computational methods applied to protein function prediction, with particular emphasis on structure-based and deep learning approaches, while highlighting their contributions and limitations in addressing AMR.

## Bacterial resistance and functional annotation

**Fig. 1:**
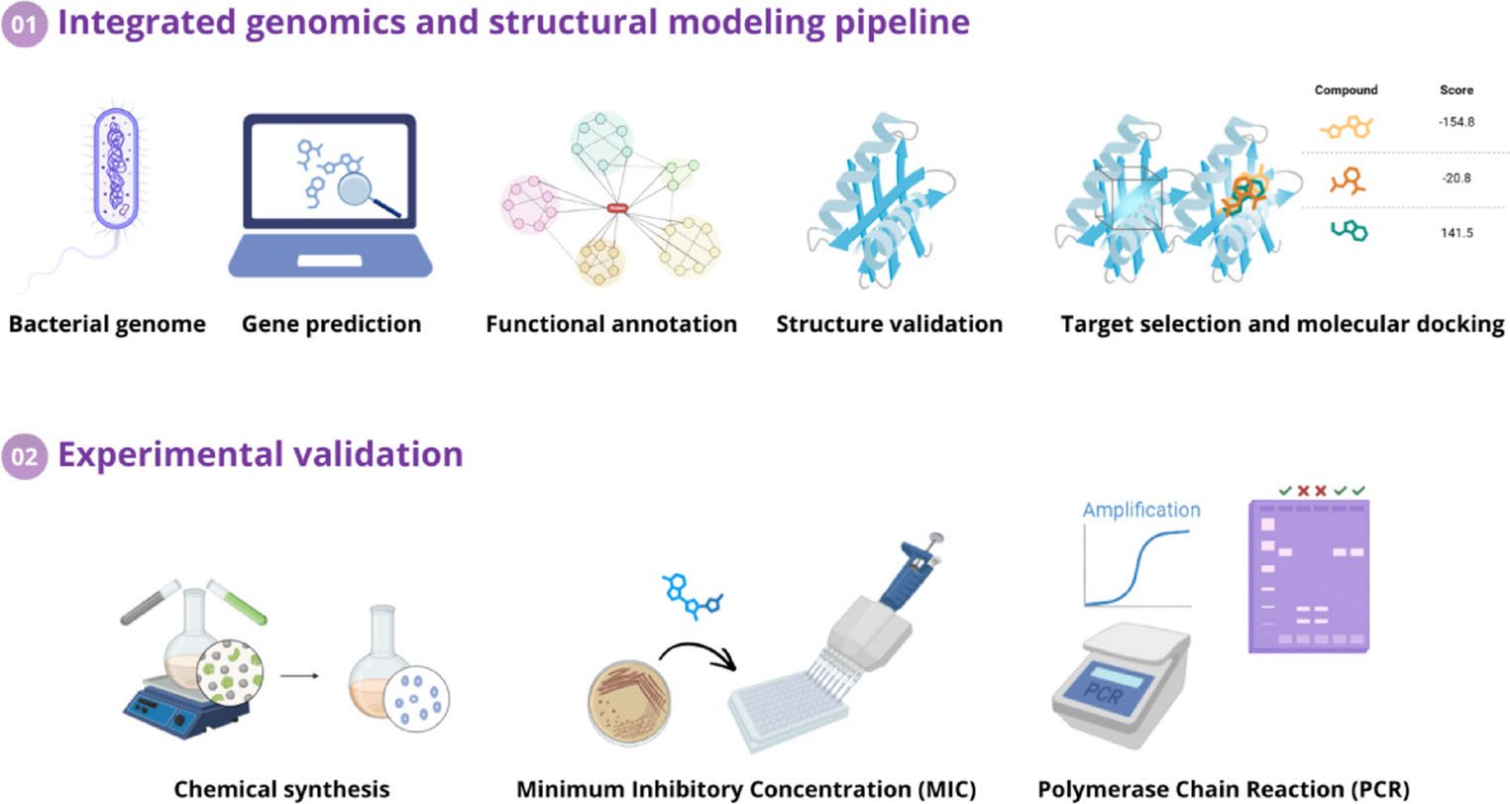
Integration of structural modeling for the functional prediction of proteins encoded in bacterial genomes associated with antibiotic resistance

Developing new antibiotics involves high financial costs and substantial time investments [10]. Bacterial pathogens have, over time, evolved multiple mechanisms of AMR, particularly in response to selective pressures [2]. As a consequence of the rapid increase in microbial resistance, the World Health Organization implemented actions to curb the inappropriate use of antimicrobials [10]. Accordingly, new strategies to combat bacterial resistance that are cost-effective and reduce development times are urgently needed [11]. Among these, bioinformatics has proven to be a dependable approach for identifying genes and molecular targets that operate under various conditions and may enhance antibiotic activity. Through in silico annotations, it is possible to predict structures, simulate molecular interactions, and more accurately select targets for subsequent in vitro and in vivo assays, thereby reducing development costs and times. From genomic data, target selection and annotation can be performed through different methodological approaches (Fig. 1).

One of the more common, rapid, and cost-effective strategies for combating bacterial resistance is illustrated in Fig. 1. This workflow begins with gene prediction from bacterial genomes, followed by functional annotation and identification of genes associated with AMR. These genes are subsequently analyzed using structural modeling tools to predict their three-dimensional protein structures, which enables the selection of more specific targets for experimental validation [12].

Integrating genomics and structural modeling thus emerges as a promising strategy, as it relies on functional annotation, and may involve either established targets, thereby enhancing their activity, or targets derived from understudied or clinically relevant microorganisms. This approach allows one to explore conserved structural elements, identify functional domains, and apply molecular docking simulations to predict ligand interactions [13].

## Structural modeling

Protein structure modeling currently is one of the best approaches in computational biology, particularly for functionally annotating proteins encoded in the genomes of multidrug-resistant bacteria [14–16]. Notable tools in this domain include AlphaFold2 [17, 18] and RoseTTAFold [19].

AlphaFold2, developed by DeepMind, represented a milestone in structural modeling by achieving near-experimental accuracy, as demonstrated during the 14th Critical Assessment of Protein Structure Prediction [17, 20–23]. Its innovative architecture, based on multi-head attention and simultaneous integration of distance and angular relationships between residues, enabled major advances in predicting structures of proteins with low homology to previously resolved structures [17, 24]. Similarly, RoseTTAFold, developed by the Baker Lab, employs a three-track neural network (sequence, structure, and multiple sequence alignments) to provide fast and accurate structure prediction, even for proteins that are challenging to model [9, 19].

The impact of such tools is particularly evident in the study of proteins with previously unknown function, frequently annotated as “hypothetical” in multidrug-resistant bacterial genomes [25, 26]. Reliable structural models for these proteins enable inference of potential biological functions based on conserved domains, active sites, or interaction interfaces, often through comparative analyses with structural databases such as CATH and SCOPe [27, 28].

It is important to emphasize, however, that structural similarity alone does not necessarily imply functional equivalence, particularly for hypothetical proteins lacking previously characterized homologs. Proteins may adopt similar overall folds while performing distinct biological functions, just as functionally related proteins may display considerable structural divergence. This is well illustrated by penicillin-binding proteins and serine β-lactamases, which share a common evolutionary origin, a conserved active-site serine-based catalytic mechanism, and an overall structural fold, yet perform markedly different biological roles: the former catalyze peptidoglycan synthesis and cell-wall maintenance, whereas the latter hydrolyze β-lactam antibiotics and represent one of the most clinically significant mechanisms of antimicrobial resistance [29].

Given this distinction between structural and functional similarity, reliable functional annotation generally requires complementary lines of evidence beyond structure prediction alone, including conserved domain analysis, active-site characterization, and assessment of evolutionary conservation across homologous sequences [30, 31]. Ultimately, experimental validation, such as enzymatic assays, site-directed mutagenesis, or structural determination by X-ray crystallography or cryo-electron microscopy, remains essential to confirm the biological function inferred from predicted structures, particularly before advancing candidate targets toward downstream stages of drug discovery.

Examples reported in the literature include the modeling of proteins encoded in the genomes of *Klebsiella pneumoniae* and *Acinetobacter baumannii*, in which various previously hypothetical proteins had structures predicted by AlphaFold2 [21, 32, 33]. These structures were subsequently employed to infer functions associated with antibiotic resistance, including enzymatic modification, efflux mechanisms, and drug inactivation [34–36]. Such predictions guide more targeted functional experiments and may accelerate the identification of novel therapeutic targets [37, 38].

Therefore, AI-driven structural modeling has revolutionized functional genomics, particularly in the context of multidrug-resistant bacteria [37, 39]. This approach supports in silico functional characterization of previously unexplored proteins, offering new insight into the molecular mechanisms of AMR and supporting developing innovative therapeutic strategies.

AI-based structural modeling provides highly accurate three-dimensional models; however, complementary computational approaches are required to investigate protein behavior and molecular interactions. In this context, molecular dynamics (MD) simulations provide insights into the dynamic behavior of proteins, complexes with bioactive molecules, and their interactions with biological targets, thereby improving the understanding of the properties, functions, and mechanisms of action or inhibition of biomacromolecules. Furthermore, they support the rational design of new therapies by simulating atomic movements over time, providing insights that complement those obtained through conventional experimental techniques [40, 41].

MD simulations enable the investigation of the temporal evolution of proteins and protein–ligand complexes by evaluating conformational flexibility, structural rearrangements, binding stability, and interaction networks. Accordingly, advances in simulation algorithms and platforms have increased computational efficiency, accuracy, and usability, thereby expanding their application in structure-based drug discovery. Additionally, these simulations refine docking poses and assist in estimating binding free energies using approaches such as MM/PBSA and MM/GBSA, thereby increasing the reliability of target prioritization and guiding the rational design of antimicrobial bioactive compounds [42, 43].

## Insights from bacterial genomics into antimicrobial resistance

The emergence of modern technologies able to analyze large volumes of biological datasets has led to rapid annual increases in bacterial protein identification. This progress represents a promising opportunity to elucidate molecular functions [44]. In the post-genomic era, functional identification is essential, given the physiological and pathological processes these proteins regulate in biological systems. Understanding protein function is therefore fundamental for uncovering potential therapeutic targets [45].

In this context, one of the biggest obstacles is the growing bacterial antibiotic resistance, which poses a major threat to global public health. Should effective interventions not be implemented, resistant infections may cause up to 10 million deaths annually by 2050. Thus, elucidating the molecular mechanisms sustaining AMR is crucial to guide preventive and therapeutic actions [46].

Although several databases are crucial for AMR surveillance, access to structural data remains vital for understanding how resistance emerges and spreads. Structure-based analyses are essential to elucidating how point mutations affect interactions with ligands (e.g., antibiotics) and how these alterations impact key cellular processes. Improving strategies to track specific mutations is therefore a key step not only in understanding the dissemination of AMR-related genes but also expanding knowledge of protein function and molecular mechanisms involved [47].

Computational approaches enable prediction of potential biological targets for prospective pharmacological compounds. Such predictions provide valuable insights into possible mechanisms of action and therapeutic outcomes [48]. However, identifying and structurally characterizing functional domains responsible for receptor interactions, particularly those recognizing lipopolysaccharides remains a largely unexplored field [33].

In addressing *A. baumannii*, a critical pathogen due to its high-level resistance, computational tools prove highly effective, offering high-precision predictions regarding complex biomolecular interactions. This capability extends to modeling of functional domains within phage tail fiber and spike proteins, enabling detailed sequence and structural analysis. Integrating these predictions allows for a deeper investigation into the molecular basis of phage-bacteria specificity, particularly concerning receptor-binding proteins [33].

## Prediction of putative proteins associated with antibiotic resistance

Prediction of putative antibiotic resistance-associated proteins currently represents a cornerstone of bioinformatics in clinical and evolutionary microbiology [49]. From raw genomic data, computational approaches enable identification of genes and proteins not yet experimentally characterized but displaying structural, functional, or evolutionary signatures consistent with resistance mechanisms [16]. This process involves integrating sequence similarity analyses, conserved domains, catalytic motifs, and genomic context, allowing inference of probable functions even where classic database annotations are absent [50].

Current pipelines combine automated annotation with AMR-specific databases, machine learning models, and phylogenomic analyses [51]. Detecting putative resistance-associated proteins is not limited to classic enzymes such as β-lactamases; it also includes efflux transporters, regulatory proteins, stress-response systems, and cell membrane remodeling components [52]. This systems-level approach is essential for exploring emerging or non-canonical resistance mechanisms.

From a translational perspective, accurate prediction of these proteins directly impacts genomic surveillance, development of new antimicrobials, and combinatorial therapy strategies [53]. Mapping the potential resistome of clinical pathogens, particularly multidrug-resistant isolates, provides critical support for anticipating resistance profiles, guiding therapeutic decision-making, and identifying promising molecular targets [54].

Genomic and bioinformatic approaches foster an integrated understanding of how bacterial genetic diversity underpins the emergence and dissemination of AMR. By linking genomic data with well-characterized molecular mechanisms, it becomes possible to identify recurring resistance patterns, prioritize genes and pathways critical for bacterial survival, and guide the development of more effective therapeutic strategies [55].

**Fig. 2:**
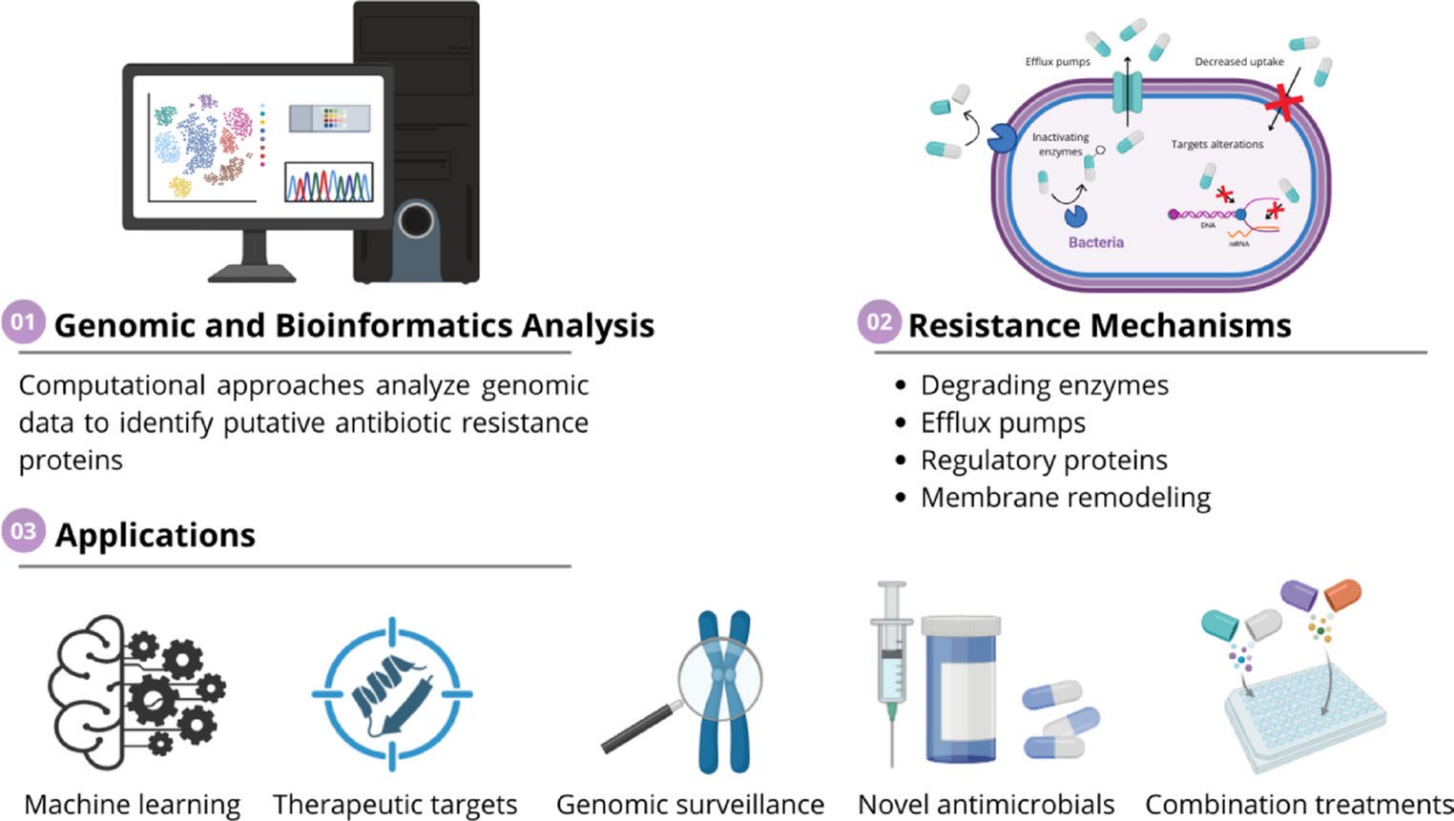
Genomic and bioinformatic analyses in identifying putative antibiotic resistance proteins from bacterial genomes. Key resistance mechanisms are highlighted, including enzymatic antibiotic degradation, efflux pumps, regulatory proteins, and membrane remodeling. The figure also summarizes major applications, such as machine learning-based predictions, target identification, genomic surveillance, new antimicrobial discovery, and development of combination therapies

Figure 2 provides an overview of genomic and bioinformatic methods in AMR research, highlighting how computational analyses support identification of proteins associated with antibiotic resistance. It summarizes the main bacterial resistance mechanisms, including enzymatic degradation, efflux pumps, regulatory proteins, and membrane remodeling, and illustrates practical applications such as genomic surveillance, therapeutic target identification, antimicrobial discovery, combination therapy strategies, and the use of machine learning to support therapeutic decision-making.

Structural modeling and computational servers play fundamental roles in predicting the three-dimensional structures of target proteins. These resources employ different approaches, such as homology modeling, ab initio prediction, and threading enable construction of reliable structural models. Obtaining such models is highly relevant for understanding protein molecular function and informing subsequent drug discovery efforts [56].

Among targets of particular interest are enzymes involved in bacterial cell wall biosynthesis are particularly noteworthy, as they perform functions essential for cellular viability and absence of direct homologs in eukaryotic organisms. Many of these enzymes have had their three-dimensional structures experimentally determined or computationally predicted, facilitating the identification of features associated with their activity. This information is widely used in molecular modeling approaches, reinforcing the central role of structural modeling in discovering and validating new antimicrobial targets [57].

One example reported in the literature is structural modeling in target discovery is the application of subtractive proteomics combined with molecular docking to identify essential proteins in *Pseudomonas aeruginosa*, including efflux pump family members associated with multidrug resistance to multiple antibiotic classes. By predicting these protein structures and simulating interactions with candidate inhibitors, researchers have identified compounds with high binding affinity, suggesting their potential as effective inhibitors and emphasizing the relevance of non-conventional targets beyond classical active sites [58].

In addition to insights into individual proteins, structural modeling enables the exploration of protein–protein interactions as promising molecular targets. Disruption of these interactions may compromise protein complexes critical for cellular survival. In Gram-negative bacteria, many such interfaces differ structurally from those in eukaryotes, favoring the development of selective therapeutic strategies and expanding antimicrobial target repertoire [59].

## Applications in *Klebsiella pneumoniae* and *Acinetobacter baumannii*

Protein structural modeling has become a strategic tool for expanding the functional understanding of genes in the genomes of *K. pneumoniae* and *A. baumannii*, particularly those annotated as hypothetical in conventional analyses [60, 61]. Both pathogens exhibit high genomic plasticity and frequently acquire mobile genetic elements, complicating function assignment based solely on sequence similarity [62, 63]. In this context, three-dimensional structure prediction enables identification of conserved folds, catalytic motifs, and functional domains not readily apparent from purely linear analyses.

In *K. pneumoniae*, structural modeling is directly applicable in the characterization of proteins associated with AMR and virulence [64]. Predicted structures for β-lactamases, efflux pump regulatory proteins, and iron acquisition factors allow identification of active sites, cofactor-binding regions, and relevant protein–protein interaction interfaces [65]. These insights clarify functional variations among alleles in different multidrug-resistant lineages. Integrating structural modeling with molecular docking analyses has enables prioritization of conserved therapeutic targets and in silico evaluations of interactions between these proteins and existing drugs or novel candidate molecules [65].

For example, structural models of β-lactamases and efflux-associated proteins have been used to map catalytic residues and ligand-binding pockets, allowing molecular docking analyses to prioritize compounds with predicted inhibitory activity before experimental validation [65]. This workflow illustrates how computational modeling can reduce the number of candidates requiring laboratory testing while providing mechanistic insights into protein–ligand interactions.

In *A. baumannii*, structural modeling is particularly relevant given the abundance of hypothetical genes and the complexity of its resistance mechanisms [66]. Proteins associated with outer membrane integrity, secretion systems, and oxidative stress responses have been better characterized through structural analyses, allowing the inference of functions related to persistence and antimicrobial tolerance [67].

Similarly, structural predictions of proteins involved in outer membrane maintenance and oxidative stress responses have enabled identification of conserved functional domains and potential druggable pockets, supporting the prioritization of essential proteins for subsequent virtual screening and experimental validation [67]. From an applied perspective, these predictions support identification of essential, conserved proteins with low homology to human proteins, reinforcing their value as potential targets for developing new antimicrobial agents [42].

**Fig. 3:**
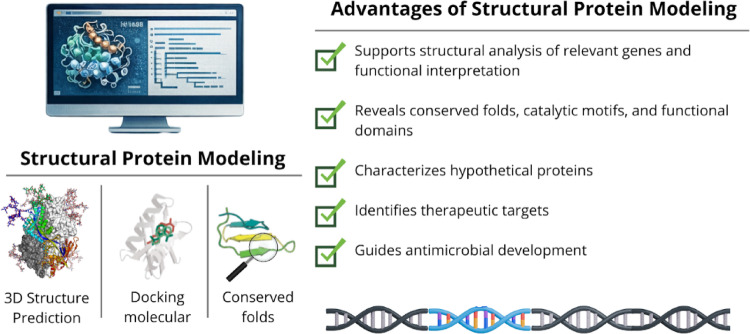
The main components and advantages of structural protein modeling in antimicrobial research. It highlights three-dimensional structure prediction, molecular docking, and identification of conserved protein folds as essential steps for functional interpretation. The advantages include characterization of hypothetical proteins, identification of targets, motif and domain analysis, and design of new antimicrobial bioactive compounds

In this context, protein structural modeling emerges as a pivotal link between genomic data and design of new bioactive compounds. Structural analysis enables functional assignment of previously uncharacterized proteins and identification of conserved regions and catalytic sites with therapeutic potential. Integrating structural prediction with molecular docking refines target selection and guides design or repurposing of antimicrobial compounds, reducing uncertainty in development and increasing the likelihood of experimental success. Figure 3 summarizes the role of protein structural modeling as a central tool in the design of new antimicrobial bioactive compounds, illustrating how three-dimensional structure prediction, fold analysis, and docking contribute to functional interpretation of relevant genes, including hypothetical proteins. The figure also emphasizes advantages in target identification and drug development, underscoring the importance of structural bioinformatics in overcoming the obstacles posed by bacterial resistance.

## Challenges and limitations

Despite significant advances, one of the main challenges of structural modeling of multidrug-resistant bacterial genomes is the scarcity of experimental data for validating in silico predictions [56]. In pathogens such as *K. pneumoniae* and *A. baumannii*, many modeled proteins still lack functional confirmation by laboratory-based methods, including X-ray crystallography, cryo-electron microscopy, enzymatic assays, or mutagenesis studies. This limitation may introduce uncertainty regarding assigned functions, as even state-of-the-art predicted structures do not fully replace experimental evidence.

An additional barrier is related to the static nature of the protein structures generated by AlphaFold and related deep learning-based tools. These models are trained mostly on experimentally determined structures deposited in the Protein Data Bank; as such structures are typically crystallized in a single, thermodynamically stable conformation, the models tend to predict a static structure rather than the broader set of conformational states that a protein may adopt. This limitation is particularly important for proteins that undergo substantial conformational rearrangements upon binding to ligands, during catalysis, or when interacting with other biomolecules, a behavior frequently observed in enzymes, transporters, and efflux pumps associated with antimicrobial resistance [68, 69].

Structures predicted by AlphaFold2 and AlphaFold3 tend to resemble the *apo* (ligand-free) state and, in several reported cases, do not reproduce ligand-induced conformational changes, cryptic binding pockets, or alternative active-site geometries, which may compromise subsequent structure-based applications such as molecular docking and virtual screening. Although AlphaFold3 has improved the modeling of protein–ligand complexes relative to previous versions, current evidence indicates that “induced-fit” conformational changes, especially in dynamic or allosteric systems, remain difficult to reproduce reliably. Overcoming this issue will likely depend on integrating AlphaFold-derived models with complementary approaches capable of exploring conformational space, such as the molecular dynamics simulations described earlier [18, 21, 70].

Another important issue is dependence on genomic sequence quality and accuracy of available structural databases. Incomplete genomes, fragmented genes, or annotation errors directly affect model reliability [71]. In highly recombinogenic organisms (e.g., *A. baumannii*), allelic diversity may result in divergent structures with distinct functions, complicating generalizations [72]. Moreover, proteins lacking close structural homologs, predicted models tend to exhibit lower accuracy, limiting their application in more refined analyses such as design of new bioactive compounds or detailed investigation of molecular mechanisms of resistance [73].

In this context, the need for integrated pipelines combining structural modeling with additional biological assays is highly evident. Integrating three-dimensional structure prediction, molecular docking, gene expression data, essentiality analyses, and genomic context enables more robust and biologically meaningful interpretations [74]. For instance, a protein that appears structurally promising as a target may not be expressed under clinically relevant conditions, diminishing its practical value.

## Future perspectives and conclusion

Recent advances in AI applications are optimizing protein engineering and expediting in silico modeling, particularly in the context of AMR. Machine learning approaches enable integration of sequence, structural, and functional data, thereby transforming functional genomics into a more automated and data-driven area. In infectious diseases research, this is especially relevant for prioritizing biologically and clinically relevant targets in multidrug-resistant pathogens [75].

Deep learning models represent a particularly promising trend, as they enable virtual screening and compound optimization by integrating functional and sequence-based data to establish structure–activity relationships. By coupling these insights with genomic information, it becomes possible to more accurately elucidate the biological roles of bacterial proteins. Modeling protein–protein and protein–ligand interactions provide important mechanistic insights for target identification and for developing inhibitors with higher specificity [76].

Examples of deep learning-based approaches include AtomNet [77], a deep convolutional neural network (DCNN) developed for structure-based drug discovery, and AlphaFold, both of which have shown highly satisfactory results in structure prediction [78]. Nevertheless, current limitations remain, many of which may be overcome by developing hybrid strategies [34, 79]. Such approaches combine structural prediction, domain analysis, molecular docking, and molecular dynamics simulations, thereby enhancing the ability to identify catalytic sites and protein–ligand interfaces relevant to AMR. Progress in this area will depend on both improvements in predictive accuracy and the interpretability of models through detailed correlation between in silico predictions and experimental validation.

Docking-based molecular structures and molecular modeling are well-established methods in drug design, and when combined with models that account for conformational flexibility and biological context, their performance can be further enhanced [80]. In this scenario, increasing the accuracy of AI tools is expected to rely on continued model training and refinement, supporting their application not only in target selection but also in protein engineering, ligand optimization, and compound design. Comparative genomics combined with bioinformatics and AI is redefining antimicrobial target identification and may, in the future, assume a central role in the development of innovative and clinically relevant therapies against resistant pathogens.

The literature reviewed indicates that genomic integration, structural modeling, and AI-based approaches play an important role in the functional characterization of proteins associated with antimicrobial resistance. Accordingly, the computational strategies discussed reduce gaps in bacterial genomics and transform large-scale omics data into interpretable biological knowledge, thereby strengthening the study of complex infectious systems. Consolidation of these computational workflows is expected to directly impact future research and antimicrobial development, particularly when complemented by experimental validation, enabling resource rationalization and reducing the time required to identify new drugs.

## Data Availability

No datasets were generated or analysed during the current study.
